# Two case reports of maturity-onset diabetes of the young type 3 caused by the hepatocyte nuclear factor 1α gene mutation

**DOI:** 10.1515/med-2023-0705

**Published:** 2023-05-12

**Authors:** Qian Wen, Yuwen Li, Huige Shao, Jun Ma, Yi Lin, Yihu Sun, Ting Liu

**Affiliations:** Graduate School, Hunan University of Chinese Medicine, Changsha, Hunan, 410208, China; Department of Endocrinology, The Affiliated Changsha Central Hospital, Hengyang Medical School, University of South China, Changsha, Hunan, 410004, China

**Keywords:** diabetes mellitus, maturity-onset diabetes of the young type 3, hepatocyte nuclear factor 1α, mutation, gene sequencing

## Abstract

Maturity-onset diabetes of the young type 3 (MODY3) is a specific type of diabetes mellitus with inherited impairment of the islet β cell function due to the mutation in the hepatocyte nuclear factor 1α (HNF1α) gene. It is a rare condition and easily misdiagnosed as T1DM or T2DM. In this study, the clinical features of two unrelated Chinese MODY3 probands were described and analyzed. Next-generation sequencing was performed to identify the mutated genes, and Sanger sequencing was employed to verify the location of the pathogenic variant in the related family members. It was found that proband 1 inherited a start codon mutation c.2T>C (p.Met1?) in exon 1 of the HNF1α gene from his affected mother, and proband 2 inherited a frameshift mutation c.1136_1137del (p.Pro379fs) in exon 6 of the HNF1α gene also from her affected mother. Proband 1 and proband 2 differed in islet dysfunction, complications, and treatments due to their different disease durations and levels of hemoglobin A_1c_ (HbA1c). The findings of this study demonstrate that early identification of MODY and diagnosis through genetic testing are critical for the treatment of the patient.

## 1 Introduction

Maturity-onset diabetes of the young (MODY) is a monogenic, autosomal, and dominantly inherited form of diabetes [1]. To date, at least 14 MODY subtypes have been identified depending on the involved gene and clinical phenotypes, and these subtypes differ in terms of the onset age, pattern of hyperglycemia, responsiveness to treatment, and extra-pancreatic manifestations. The most prevalent gene mutations are MODY1-HNF4α, MODY2-GCK, and MODY3-HNF1α [2].

Among different subtypes of MODY, MODY3 was found to be associated with mutations in the hepatocyte nuclear factor 1α (HNF1α) gene. The prevalence of MODY3 in the general population ranges from 0.02 to 0.04% (the prevalence in Caucasians was higher than in the Asian population) [3]. HNF1α is a homeodomain-containing transcription factor regulating the expression of pancreas-specific genes. Mutations in HNF1α affect proteins such as glucose transporter 2, amylin, insulin, and l-pyruvate kinase, associated with insulin secretion and glucose metabolism [4]. Currently, misdiagnosis, improper treatment, poor blood sugar control, and even complications occur due to the overlap between MODY3 patients and T1DM/T2DM patients in terms of age of onset, family history, and treatment methods, as well as the lack of understanding of MODY3 by clinicians. The diagnosis of MODY3 relies on genetic testing, a crucial tool for defining the etiology, implementing appropriate and individualized care, formulating accurate prognoses, predicting complications, and providing adequate family counseling.

In this article, a start codon mutation and a frameshift mutation of the HNF1α gene in two unrelated Chinese families were reported. Based on the results of genetic testing, we facilitated precise diagnosis and modified appropriate treatment.

## 2 Case presentation

### 2.1 Case 1

**Figure 1:**
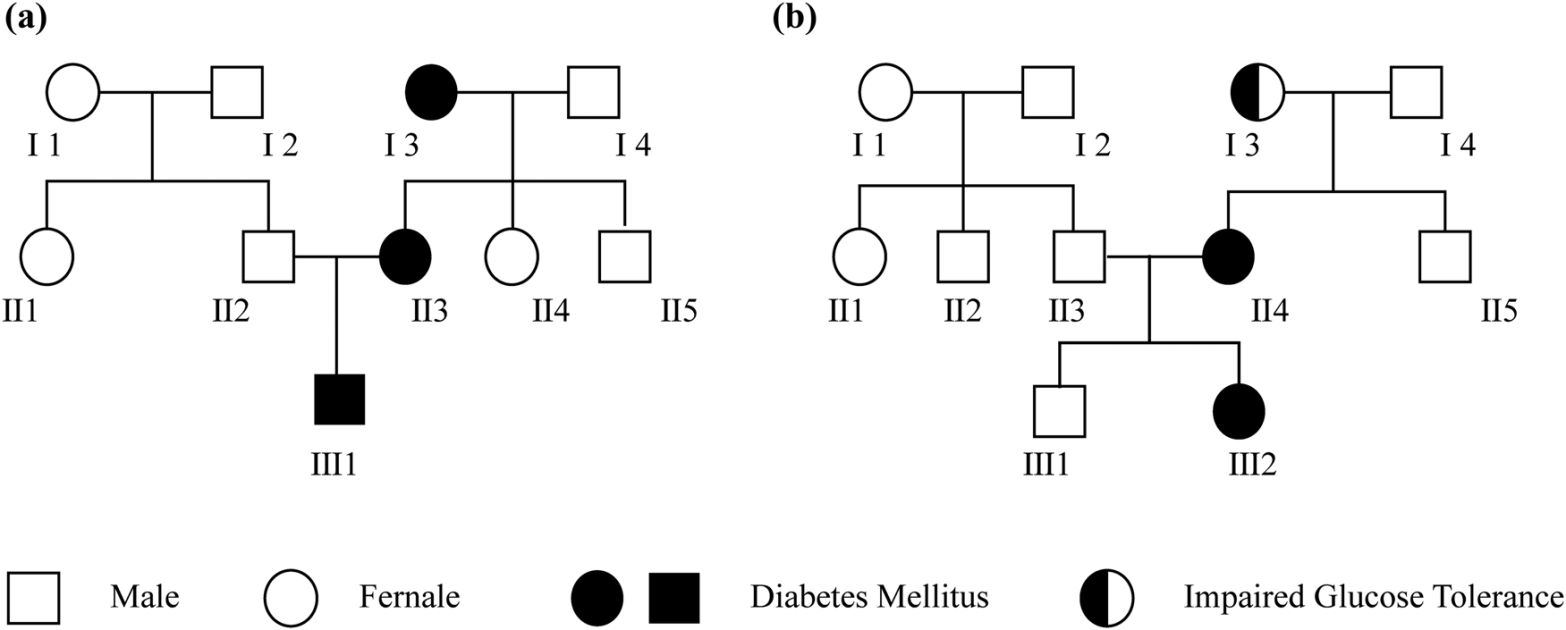
(a) Proband 1’s family diagram. I 3 – grandmother with T2DM diagnosed at the age of 51 years, medicated with slow-acting insulin; II 3 – mother with T2DM diagnosed at the age of 39 years, medicated with glimepiride 4 mg once a day; III 1 – MODY3 diagnosed at the age of 23 years. (b) Proband 2’s family diagram. I 3 – grandmother with impaired glucose tolerance diagnosed at the age of 55 years, treated with diet and exercise; II 4 – mother with T2DM diagnosed at the age of 41 years, treated with diet and exercise; III 2 – MODY3 diagnosed at the age of 19 years.

**Figure 2:**
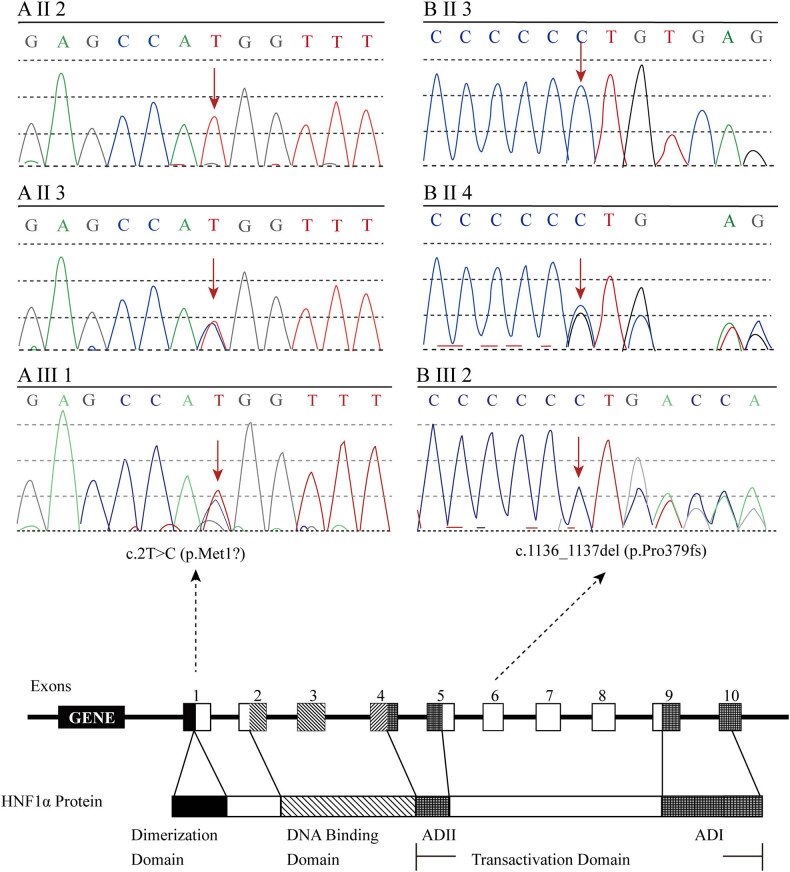
The sequencing chromatogram and position of the mutation in HNF1α gene. (A Ⅲ 1 and A Ⅱ 3) Proband 1 and his mother presented the same mutation c.2T > C (p.Met1?) in the start codon in exon 1 of the HNF1α gene. (B Ⅲ 2 and B Ⅱ 4) Proband 2 and her mother presented the same frameshift mutation c.1136_1137del (p.Pro379fs) in exon 6 of the HNF1α gene. Arrows indicate the mutation site.

A 23-year-old Chinese male was admitted to Changsha Central Hospital (Hunan, China) on July 5, 2018, due to hyperglycemia revealed by a routine examination performed 6 months earlier. He had no symptoms of diabetes such as polydipsia, polyuria, overeating, and weight loss. The proband’s mother and maternal grandmother had already been diagnosed with diabetes (Figure 1). Physical examination showed a blood pressure of 110/70 mmHg and a body mass index (BMI) of 18.1 kg/m^2^. No abnormalities were indicated in cardiopulmonary and abdominal examinations. Bilateral dorsalis pedis pulsed normally. The laboratory examination results were as follows: fasting blood glucose (FBG): 6.82 mmol/l; 2 h-postprandial blood glucose (PBG): 11.12 mmol/l; fasting C-peptide: 0.38 nmol/l; 2 h-postprandial C-peptide: 0.97 nmol/l; hemoglobin A_1c_ (HbA1c): 6.80%; and urine glucose: 4+. The diabetic autoimmune antibodies were negative. Besides, no abnormal results in biochemical examination, urinary microalbumin creatinine ratio (UACR), abdominal B-ultrasound, electroneuromyography, or fundus screening were detected (Table 1). Based on the proband’s medical history, physical examinations, and laboratory evaluations, he was initially suspected with MODY. Therefore, next-generation sequencing was performed to identify the mutated genes, and Sanger sequencing was used to verify the location of the pathogenic variant in the related family members. Gene sequencing revealed that the proband and his mother presented the same mutation *c.2T>C (p.Met1?)* in the start codon in exon 1 of the HNF1α gene but not his father (Figure 2).

**Table 1:** Clinical characteristics of proband 1 and proband 2

Variables	Proband 1	Proband 2	Normal range
Gender	M	F	/
Age at onset (years)	23	12	/
WHR	0.89	0.96	/
BMI (kg/m^2^)	18.1	16.7	18.5–23.9
FBG (mmol/l)	6.82	8.98	3.89–6.11
2 h PBG (mmol/l)	11.12	20.37	<7.8
FCP (nmol/l)	0.38	0.23	0.16–1.68
2 h CP (nmol/l)	0.97	0.31	0.16–1.68
HbA1c (%)	6.8	14.4	3.6–6.0
Triglycerides (mmol/l)	1.59	1.64	0.55–1.70
Total cholesterol (mmol/l)	4.10	4.98	3.40–5.20
LDL-C (mmol/l)	2.48	2.76	1.50–3.00
HDL-C (mmol/l)	1.59	1.44	0.78–2.00
ALT (U/l)	12	25	7–40
AST (U/l)	17	22	13–35
Albumin (g/l)	50	43	40–55
Creatinine (μmol/l)	60	37	41–73
Uric acid (μmol/l)	236	222	155–357
Serum ketone (mmol/l)	Normal	Normal	Normal
U-GLU	4+	4+	NEG
U-PRO	NEG	1+	NEG
UACR (mg/g)	25	417.8	<30
TSH (mIU/l)	1.219	2.484	0.350–4.940
Serum electrolyte	Normal	Normal	Normal
Diabetic autoimmune antibodies	NEG	NEG	NEG
Pancreas/kidney dysplasia	N/A	N/A	/
Complications	/	DPN, DN	/

F, female; M, male; BMI, body mass index; WHR, waist/hip ratio; FBG, fasting blood glucose; PBG, postprandial blood glucose; FCP, fasting C-peptide; 2 h CP, 2 h-postprandial C-peptide; HbA1c, hemoglobin A_1C_; HDL-C, high-density lipoprotein cholesterol; LDL-C, low-density lipoprotein cholesterol; ALT, alanine transaminase; AST, aspartate aminotransferase; U-GLU, urine glucose; U-PRO, urine protein; UACR, urinary microalbumin/creatinine ratio; TSH, thyroid-stimulating hormone; diabetic autoimmune antibody, including GADA, IAA, ICA, and ZnT_8_; DPN, diabetic peripheral neuropathy; DN, diabetic nephropathy.

For the treatment, the proband was first misdiagnosed as T2DM in the community hospital and treated with metformin. However, this therapy appeared to be useless for controlling blood glucose within the optimal range. After diagnosis in our hospital, metformin was discontinued and gliclazide sustained-release tablet, 30 mg once a day, was prescribed. During a long follow-up, the proband’s blood glucose control was satisfactory. Currently, he is still receiving gliclazide sustained-release tablet at the same dosage, and his FBG, 2 h-PBG, and HbA1c levels are controlled at 4.5–6.0 mmol/l, 6.8–7.5 mmol/l, and 6.10%, respectively. His impaired islet function shows no serious progression, and there is no occurrence of diabetes complications.

### 2.2 Case 2

A 19-year-old Chinese female was admitted to our hospital on January 6, 2020, complaining of hyperglycemia for 7 years and limb numbness for 2 years. A routine examination performed 7 years earlier revealed elevated FBG and PBG levels. She had obvious polydipsia and polyuria but refused any treatment to control the blood glucose. Two years ago, limb numbness gradually appeared, especially in the distal end. Her mother and grandmother had abnormal blood glucose in the past (Figure 1). Physical examination showed a blood pressure of 124/82 mmHg and a BMI of 16.7 kg/m^2^. No abnormalities were indicated in cardiopulmonary and abdominal examinations. Bilateral dorsalis pedis pulsed normally. The results of laboratory examination were as follows: FBG: 8.98 mmol/l; 2 h-PBG: 20.37 mmol/l; fasting C-peptide: 0.23 nmol/l; 2 h-postprandial C-peptide: 0.31 nmol/l; HbA1c: 14.4%; total cholesterol: 4.98 mmol/l; urine glucose: 4+; and urine protein: 1+. The diabetic autoimmune antibodies were negative. The UACR was 417.8 mg/g. Electroneuromyography showed severe and multiple damages in the peripheral nerve of limbs. Biochemical examination and abdominal ultrasound or fundus photography revealed no abnormalities. Color Doppler ultrasound examination showed that her lower extremity arteries were not atherosclerotic (Table 1). Gene sequencing indicated that the proband and her mother presented the same frameshift mutation *c.1136_1137del (p.Pro379fs)* in exon 6 of the HNF1α gene, but not her father (Figure 2). Therefore, the proband was diagnosed as MODY3 with diabetic peripheral neuropathy and diabetic nephropathy (G2A3).

For the treatment, the proband was diagnosed as T2DM and diabetic peripheral neuropathy in the community hospital 2 years earlier. She was initially prescribed with acarbose and metformin to control blood glucose but achieved poor blood glucose control. After confirming the diagnosis in our hospital, gliclazide sustained-release tablet 90 mg was applied once a day, but her blood glucose was still poorly controlled. Therefore, insulin therapy was prescribed. Specifically, predormital insulin (recombinant insulin glargine) was administered by subcutaneous injection at 15 U once a day, and preprandial insulin (insulin lispro) was administered 5 min before breakfast (4 U), lunch (5 U), and dinner (5 U), respectively. According to her diabetic complication, this therapy was further supplemented with the treatment of nerve nourishing, microcirculation improvement, and urinary protein reduction. Currently, her blood glucose level fluctuates within a reasonable range, and her HbAlc level is maintained at 6.9% with insulin therapy. There is no significant progression in complications.


**Ethics approval and consent to participate:** The present study was approved by The Affiliated Changsha Central Hospital, Hengyang Medical School, University of South China (approval no: 2017-S023). The patients provided written informed consent for the publication of individual clinical details and all the accompanying images.

## 3 Discussion and conclusions

MODY3 is a monogenic disease with autosomal-dominant inheritance due to HNF1α haplo-insufficiency or dominant-negative effects. The HNF1α gene is located on chromosome 12 in the region 12q24.2 [5]. The mutations in the HNF1α gene are distributed in the promoter and across ten exons, with over 80% of the mutations occurring in exons 1–6. More specifically, mutations observed in HNF1α exons 2 and 4 are the most, while those observed in exons 5 and 10 are the least. To date, several variants have been reported, including missense, frameshift, nonsense, splicing mutations, in-frame deletions, insertions and duplications of amino acids, and partial or whole-gene deletions [6]. The HNF1α protein consists of 631 amino acids, including the dimerization domain, the DNA-binding domain, and the transactivation domain [7]. In our cases, the two probands were diagnosed as MODY3 by genetic testing. Proband 1 had a start codon mutation c.2T>C (p.Met1?) located in the dimerization domain, which affected the initiation of protein translation, leading to the loss of initial amino acid sequence and protein dysfunction. Proband 2 had a frameshift mutation c.1136_1137del (p.Pro379fs) located in the transactivation domain, which affected the transactivation activity and nuclear localization. Consequently, the proline at position 379 was replaced by arginine and frameshift occurred, resulting in premature termination of translation, loss of subsequent amino acid sequence, truncation of the encoded protein, and eventually loss of the normal function.

HNF1α is mainly expressed in the pancreas, kidneys, and intestine. In β-cells of the islet, HNF1α mutations can reduce β-cell proliferation and affect the biological processes including glucose transport, glucose metabolism, and glucose-stimulated ATP production, which will activate ATP-sensitive channels and attenuate depolarization of cell membranes and internal flow of calcium ion, and thus reduce insulin secretion [8]. In the kidney, HNF1α directly regulates the expression of sodium-dependent glucose transporter 2 (SGLT-2). HNF1α mutations can reduce the SGLT-2 level, resulting in a lowered renal glucose threshold [9]. Therefore, positive urine glucose is usually observed in the early stage of MODY3 patients with normal blood glucose. In this study, the FBG level of the two patients was normal in the early stage of the disease, but the 2 h-PBG of oral glucose tolerance test was significantly elevated with strong positive urine glucose. Alongside the development of the disease, FBG gradually increases, and diabetes symptoms such as polyuria, polydipsia, and polyphagia occur; however, ketosis is rarely developed [10]. Patients with MODY3 may have microvascular complications that are comparable to T2DM patients, which are associated with poor glycemic control and longer disease duration [11]. Therefore, adequate glycemic control is critically important. Proband 1 had a shorter course of the disease. Once diagnosed, the drug of choice was prescribed for treatment immediately and the blood glucose was well controlled, without developing diabetes symptoms and complications. In comparison, Proband 2 had a 7-year course of disease with poorly controlled glucose. As the disease continued to progress, her islet secretion function was seriously damaged, and diabetes symptoms gradually emerged, complicated with diabetic peripheral neuropathy and diabetic nephropathy.

Sulfonylureas, as the preferred treatment for patients with MODY3, can specifically bind the SUR1 subunit of ATP-dependent potassium channels (KATP) and stimulate insulin secretion [12]. Sulfonylureas may allow for the withdrawal of insulin in patients misdiagnosed with T1DM without exposing them to the risk of ketoacidosis. It has been reported that part of the MODY3 patients with HNF1α mutations, such as c.1522 g>A, C.618 g>A, and C.376C>G variations, have poor response to sulfonylureas [13,14]. Besides, some patients might achieve well-controlled blood glucose with no hypoglycemic episodes when treated with glucagon-like peptide-1 receptor agonist (GLP-1RA) and dipeptidyl peptidase-IV (DPP-IV) inhibitor [15]. Following the decrease in endogenous insulin secretion, many patients would encounter progressively deteriorated glycemic control, thereby requiring insulin treatment [16]. In this study, proband 1 had acceptable islet secretion function due to a shorter course of the disease and satisfactory glucose control; thus, low-dose sulfonylurea remained effective in controlling his blood glucose. However, proband 2 had severely impaired islet secretion function due to a longer course of the disease and poor glucose control, complicated with insensitivity to sulfonylurea therapy; thus, insulin therapy was indispensable in her treatment. The different therapeutic choices for MODY3 patients were attributed to multiple factors including disease duration, glucose control, genetic variation location, and type. When newly diagnosed MODY3 patients are initially treated with sulfonylurea, intensive blood glucose monitoring is required to avoid hypoglycemic episodes. Most mutations of MODY3 are sensitive to sulfonylureas, but it seems no specific type of mutation or exon mutation is more suitable than others for sulfonylureas. Thus, further investigations are needed.

At present, the main obstacle to the diagnosis of MODY lies in the lack of in-depth understanding and clinical suspicion, and the high cost of genetic testing. In the present study, we first detailed two heterozygous variants (c.2T>C and c.1136_1137del) in HNF1α, along with clinical manifestations, diagnosis processes, treatments, and follow-up of two patients with MODY3 in two independent Chinese families. The two patients and their families were initially misdiagnosed as T2DM and were diagnosed as MODY3 after genetic testing. These two mutations are rare, and no detailed description of patients with the same mutations has been reported until our study, which highlights our novelty. Based on these two clinical cases, we emphasized the importance of early recognition of the disease signs and symptoms, which would help improve the awareness of MODY. In clinical practice, stringent screening criteria should be established to achieve a balance between cost-effectiveness and identification. For example, the results of pancreatic autoantibodies, the family history of diabetes, the age of onset, and endogenous insulin secretion should be taken into account to determine whether genetic testing is required. Once suspicion is raised, genetic testing should be performed to support the diagnosis of MODY, allowing early treatment and accurate family screening [17]. Clinically, further studies are needed to find possible biomarkers of MODY.
